# Morphoanatomical and biochemical factors associated with rice resistance to the South American rice water weevil, *Oryzophagus oryzae* (Coleoptera: Curculionidae)

**DOI:** 10.1038/s41598-022-27080-3

**Published:** 2022-12-28

**Authors:** Juliano de Bastos Pazini, José Francisco da Silva Martins, Keilor da Rosa Dorneles, Rosane Lopes Crizel, Fernando Felisberto da Silva, Fábio Clasen Chaves, Juliana Aparecida Fernando, Leandro José Dallagnol, Enio Júnior Seidel, Michael Joseph Stout, Anderson Dionei Grützmacher

**Affiliations:** 1 Department of Plant Protection, Federal University of Pelotas, Pelotas, RS 96160-000 Brazil; 2 Embrapa Temperate Agriculture, Brazilian Agricultural Research Corporation, Pelotas, RS 96010-97 Brazil; 3 Department of Agroindustrial Science and Technology, Federal University of Pelotas, Pelotas, RS 96160-000 Brazil; 4 Federal University of Pampa, São Gabriel Campus, São Gabriel, RS 97307-020 Brazil; 5 Department of Botany, Federal University of Pelotas, Pelotas, RS 96160-000 Brazil; 6 Department of Statistics, Federal University of Santa Maria, Santa Maria, RS 97105-900 Brazil; 7 Department of Entomology, Louisiana State University Agricultural Center, Baton Rouge, LA 70803 USA; 8 Present Address: Department of Entomology and Acarology, University of São Paulo, Piracicaba, SP 13418-900 Brazil

**Keywords:** Entomology, Biotic, Herbivory, Plant breeding

## Abstract

The rice water weevil, *Oryzophagus oryzae* (Coleoptera: Curculionidae), is an economically important pest of flooded rice paddies throughout South America, and species with similar life histories are present in many rice-producing regions globally (collectively referred to here as RWWs). Plant resistance is a key strategy for management of RWWs; however, the mechanisms responsible for rice resistance to RWWs are poorly understood. We investigated morphoanatomical and biochemical plant traits potentially involved in rice resistance to *O. oryzae*. Resistance-associated traits were characterized in two cultivars, ‘Dawn’ (resistant) and ‘BRS Pampa CL’ (‘Pamp’ = susceptible), which were selected from among six cultivars on 2-year field screenings. Anatomical and morphological traits of leaf tissues from ‘Pamp’ and ‘Dawn’ were similar, which perhaps explains the lack of antixenosis during host plant selection. However, significant antibiosis effects were found. The activities of antioxidant enzymes involved in plant defense, as well the content of hydroxycinnamic and hydroxybenzoic acids derivatives and lignin, were higher in roots of ‘Dawn’ than in ‘Pamp’, over the period of larval infestation in the field. Additionally, ‘Dawn’ exhibited a root sclerenchyma arranged in three layers of lignified cells, which differed from the arrangement of cells in ‘Pamp’, regardless of larval infestation. Our results provide the first evidence for specific resistance-related traits associated with mortality and malnutrition of RWWs in rice.

## Introduction

Rice (*Oryza sativa* L.) is the primary food source for more than half of the world’s population, and hence this cereal is among the most important crops for global food security. Attack by several insects constitutes one of the main constraints on the productivity of rice paddies^1^. Approximately 1,100 species of insects have been reported as pests on rice^2^. Rice water weevils (collectively referred to as RWWs) (Coleoptera: Curculionidae), which include *Lissorhoptrus oryzophilus* (Kuschel) in the Americas, Asia, and Europe^3–5^, *Lissorhoptrus brevirostris* (Suffrian) in Central America^6^, and *Oryzophagus oryzae* (Costa Lima) in South America^7^, are highly harmful to flooded rice paddies, causing up to 30% yield loss.

In Southern Brazil, the region that accounts for 92% of rice produced in Brazil^8,9^, *O. oryzae* is a widespread and chronic problem on over 65% of the cultivated rice area^10^. The use of synthetic insecticides, mainly as seed treatments, has been the most widely adopted control method for RWWs in Brazil. Although seed treatment insecticides are effective, the dependence on chemical control increases production costs and can potentially have negative impacts on the agroecosystem^11^.

Host plant resistance, which is a sustainable strategy for pest suppression^12^, stands out as an important component of integrated management programs for RWWs worldwide^13,14^. The effects of resistant host plants on arthropods are broadly classified as antixenosis (or non-preference) and antibiosis^15^; tolerance, a third category of plant resistance, does not disrupt the behavior or biology of herbivores, but rather refers to the ability of a plant to withstand herbivory without decline in yield or quality^15,16^. Antixenosis and antibiosis against RWW species have been identified in various genotypes of rice^17–21^.

The mechanisms responsible for plant resistance include plant traits that directly affect insect performance and involve physical, morphological, or chemical factors that are constitutively present in the plant or are induced in response to herbivory. Induced responses usually involve modification and accumulation of specialized plant metabolites^22^. Smith and Clement^23^ reported that direct defenses in plant tissue, which make plants repellent or toxic to herbivores, include structural barriers, such as stiffness, thickening, trichomes, and epidermal factors. Direct defenses may also include the production of allelochemicals or resistance-related proteins with antinutritional properties, such as inhibitors of digestive enzymes, lectins, alkaloids, terpenoids, and phenolic compounds.

Some of the plant traits underlying antixenosis and antibiosis against arthropod pests have been described^24^. Rice cultivars in particular have been selected based on structural and epidermal anatomical traits considered inappropriate for the feeding and oviposition of stem borer species^25^. In addition, plants respond to oxidative stress and the production of reactive oxygen species (ROS) caused by herbivore feeding by activating antioxidant enzymes^26^. Antioxidant enzymes have been reported to play an essential role in rice resistance to insect pests^27–29^. Enzymatic products can also strengthen the structure of the cell wall and reduce the nutritional value and digestibility of plant tissues, thereby reducing herbivore growth and development^12,30^.

Studies examining the resistance of rice cultivars to various RWW species have been conducted for over 50 years. Some cultivars have been shown to support low larval infestations (e.g., ‘Dawn’)^17,31^, but little knowledge has been gained about the reaction of resistant plants to the colonization, feeding, and development of these insects. To date, no studies have elucidated the morphological or chemical rice defense factors that regulate antixenosis or antibiosis to RWWs. The lack of understanding of resistance mechanisms is probably one of the main factors responsible for the decreasing level of resistance to *O. oryzae* in cultivars released by breeding programs, which aim primarily to increase yield potential and grain quality, without exploring target traits for insect resistance^32,33^. For instance, only one commercial rice cultivar has been characterized as resistant to *O. oryzae* in Brazil and this cultivar was registered more than 20 years ago^32^. Thus, identifying rice cultivars that are resistant to RWWs and determining factors responsible for resistance would represent an advance in knowledge with significant practical benefits^14,20,34^.

Here, we investigated morphoanatomical and biochemical traits that are potentially involved in rice resistance to *O. oryzae*. Our study investigated preference and performance of RWW under natural infestations in six rice cultivars in field screenings. Moreover, investigations of morphoanatomical and biochemical defense factors were conducted on two rice cultivars with contrasting susceptibility. The present study was conducted to shed light on the effects of rice resistance on RWWs, which will be helpful for the development of elite rice cultivars with resistance to RWWs by rice breeding programs.

## Results

### Field screenings

#### Feeding, oviposition, and performance of RWW

The results indicated none of the six cultivars evaluated exhibited antixenosis for feeding and oviposition by RWW adults in the 2-year field experiment (Table 1). ANOVA did not show significant differences among cultivars for the number of feeding scars (df = 5, F = 2.36, *P* = 0.08) or eggs (df = 5, F = 0.59, *P* = 0.71) counted at 5, 8 and 11 DAF (Table 1).

**Table 1:** Densities and performance variables of South American rice water weevil *Oryzophagus oryzae* on six rice cultivars in a 2-year field screening for antixenosis and antibiosis.

Cultivar^1^	Variables^2^					
	Number of feeding scars^a^	Number of eggs^b^	Number of larvae^c^	Weight of larvae (mg)^d^	TE50% (days)^e^	Weight of adult offspring (mg)^f^
ATAL	14.75 a	9.69 a	6.59 d	5.10 b	26.83 a	2.11 b
FIRM	14.58 a	8.03 a	4.67 e	4.58 c	26.83 a	2.07 b
LIGE	12.41 a	8.84 a	8.49 c	7.04 a	18.50 b	2.23 a
PAMP	12.66 a	8.82 a	14.11 a	7.34 a	18.50 b	2.29 a
QUER	13.25 a	8.06 a	9.95 b	7.29 a	16.83 b	2.25 a
DAWN	14.83 a	9.93 a	4.51 e	4.47 c	30.33 a	2.04 b
df	5	5	5	5	5	5
F	2.36	0.59	79.13	61.10	11.63	13.26
*P*	0.08	0.71	< 0.001	< 0.001	< 0.001	< 0.001
CV%	12.79	33.28	12.47	7.32	17.78	3.29

^1^Rice cultivars: ATAL = BRS Atalanta; FIRM = BRS Firmeza; LIGE = BRS Ligeirinho; PAMP = BRS Pampa CL; QUER = BRS Querência; DAWN = Dawn.

^2^Variables: ^a^ = number of feeding scars; ^b^ = number of eggs [both averages from 5, 8, and 11 days after flooding (DAF)]; ^c^ = number of larvae; ^d^ = weight of larvae (mg) (both averages from 15, 25, and 35 DAF); ^e^ = time (days) required to reach 50% adult population (TE50%); ^f^ = weight of offspring adults (mg) (average of male and female). Average values followed by the same letter does not differ significantly by the Scott Knott test (*P* < 0.05).

Antibiosis effect against RWWs varied among the six rice cultivars in the 2-year field experiment (Table 1). The ANOVA found differences among the cultivars with respect to the number (df = 5, F = 79.13, *P* ≤ 0.001) and weight (df = 5, F = 61.10, *P* ≤ 0.001) of larvae, TE50% (df = 5, F = 11.63, *P* ≤ 0.001), and weight (df = 5, F = 13.26, *P* ≤ 0.001) of adult offspring (Table 1).

**Figure 1:**
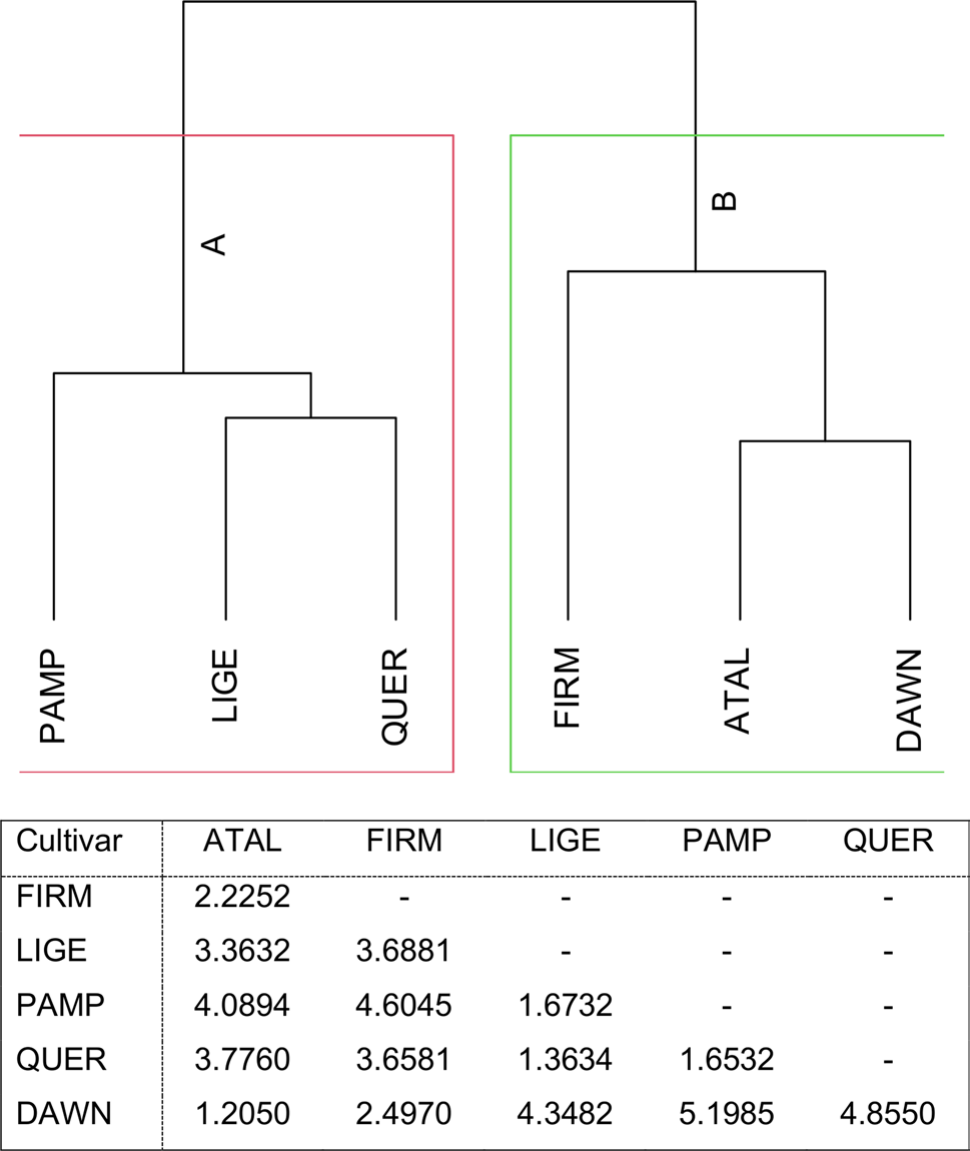
Dendrogram and similarity matrix calculated based on Euclidean distances for rice resistance to the South American rice water weevil, *Oryzophagus oryzae,* among six rice cultivars in a 2-year field screening. Rice cultivars: ATAL = BRS Atalanta; FIRM = BRS Firmeza; LIGE = BRS Ligeirinho; PAMP = BRS Pampa CL; QUER = BRS Querência; DAWN = Dawn. Variables: numbers of feeding scars and eggs [average from 5, 8, and 11 days after flooding (DAF)], number and weight of larvae [average from 15, 25, and 35 DAF], time to 50% emergence [TE50%], and weight of adult offspring [average between male and female].

The dendrogram based on densities and performance variables of RWW on the six rice cultivars revealed two groups (A and B) (Fig. 1). Cluster A consisted of ‘BRS Pampa CL’, ‘BRS Querência’, and ‘BRS Ligeirinho’, which shared characteristics distinguishing them as the most suitable hosts (Table 1), and Cluster B contained ‘BRS Atalanta’, ‘BRS Firmeza’, and ‘Dawn’, which were considered less suitable hosts for *O. oryzae* (Table 1). Distance coefficients among rice cultivars using Euclidean geometry are shown in Fig. 1. ‘Dawn’ was the cultivar with the highest average Euclidean distance from the group of suitable hosts (group A), showing a higher degree of resistance. In this sense, ‘Dawn’ and ‘BRS Pampa CL’ had the most divergent effects with an Euclidean distance of 5198 (Fig. 1), and therefore, BRS Pampa CL was categorized as susceptible to *O. oryzae* attack.

### Defense-associated traits in contrasting rice cultivars

#### Morphoanatomical traits

**Figure 2:**
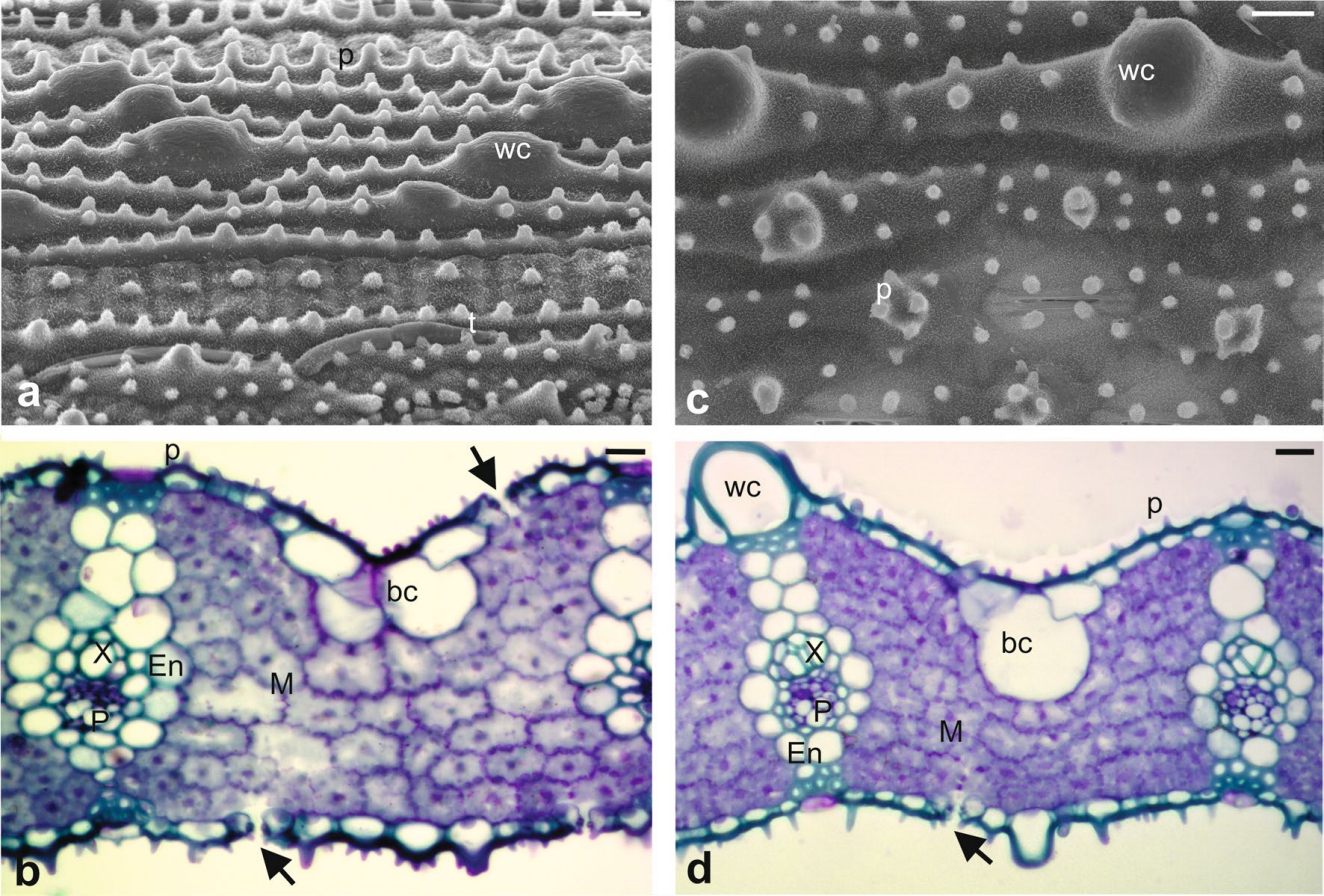
Leaf anatomy of rice cultivars resistant [‘Dawn’ = (**a**), (**b**)] and susceptible [(‘BRS Pampa CL’ = (**c**), (**d**)] to the South American rice water weevil *Oryzophagus oryzae* at 25 days after flooding (DAF). (**a**), (**c**) Scanning electron microscopy of the adaxial surface. (**b**), (**d**) Light microscopy (cross-section). M = mesophyll (mesophyll with chlorenchyma cells arranged densely packed); t = non-glandular trichome; p = papillae; wc = wart-like protuberant cell; bc = bulliform cells; X = xylem; P: phloem; En = endodermis; Arrows indicate stomata. Scale bars = (**a**) 10 µm [1000 ×], (**b**) 10 µm [1200 ×], (**c**), (**d**) 200 µm.

Leaf morphological traits of ‘Dawn’ and ‘BRS Pampa CL’ are shown in Fig. 2. The analysis indicated a high degree of similarity between the two cultivars, which contained a high concentration of silica bodies, characterized by papillae and wart-like protuberances on the adaxial surface (Fig. 2a,c). ‘Dawn’ visually had a greater concentration of silica bodies, but they were structurally smaller than those on ‘BRS Pampa CL’ (Fig. 2a), as evidenced by the comparison with the large structures of the wart-like protuberances in ‘BRS Pampa CL’ (Fig. 2c); i.e., a higher concentration of papillae was found in ‘Dawn’, but the wart-like protuberances were larger in ‘BRS Pampa CL’. Low presence of non-glandular trichomes was also observed in both cultivars, and trichomes were short, narrow, and oriented parallel to the leaf veins. No glandular trichomes were found in either cultivar (Fig. 2a,c).

Regarding the anatomical evaluation of leaf tissues, there were no apparent differences among cultivars (Fig. 2b,d). The parenchymal cells were homogeneous and densely distributed throughout the mesophyll, where few intercellular spaces were found and the bundle sheath was surrounded the vascular bundles. A slight difference was observed only for bulliform cells, which were larger in ‘BRS Pampa CL’ (Fig. 2d) than in ‘Dawn’ (Fig. 2b). Wart-like protuberant bodies were anatomically detected and appeared larger in ‘BRS Pampa CL’ (Fig. 2d).

**Figure 3:**
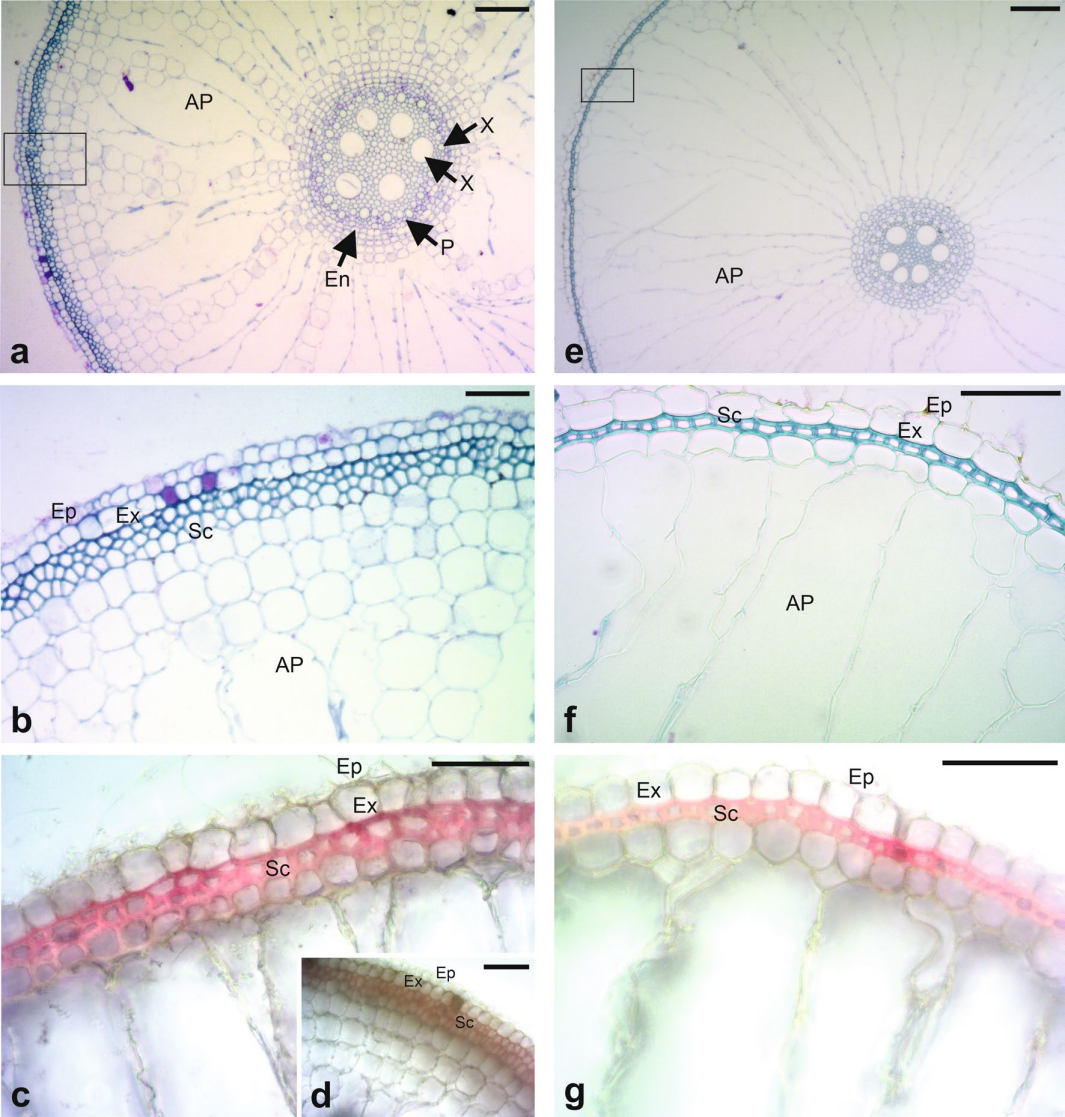
Root anatomy of rice cultivars resistant [‘Dawn’ = (**a**–**d**)] and susceptible [(‘BRS Pampa CL’ = (**e**–**g**)] to the South American rice water weevil *Oryzophagus oryzae* at 25 Days After Flooding (DAF). (**a**), (**e**) Overview, box showing the differentiation among root layers; (**b**), (**f**) Sclerenchyma cells arranged into 3-layers and 1-layer in the resistant and susceptible cultivars, respectively; (**c**), (**d**) and (**g**) Lignin detection in sclerenchyma cells by histochemical methods (red color) [(**c**) ‘Dawn’ infested plant and (**d**) ‘Dawn’ non-infested plant]. AP = aeriferous parenchyma or aerenchyma; Ep = epidermis; Ex = exodermis; En = endodermis; Sc = sclerenchyma; X = xylem; P: phloem Bars: 200 µm.

The anatomical evaluation of root tissues showed visible structural anatomical differences between the cultivars susceptible and resistant to RWW attack (Fig. 3a,b,e,f), regardless of the larval infestation of the plants at 25 DAF (roots of infested or non-infested plants). The roots of ‘BRS Pampa CL’ possessed sclerenchymal cells arranged in a thin layer. A well-developed cortex formed by vigorous aerenchyma or intercellular spaces was also observed (Fig. 3e,f). Conversely, the roots of ‘Dawn’ exhibited a sclerenchyma arranged in three layers of cells, a configuration substantially distinct from ‘BRS Pampa CL’. A slightly less developed cortex was also observed in ‘Dawn’, which exhibited more compact parenchymal cells around the aerenchyma, and which, as a result, had smaller intercellular spaces (Fig. 3a,b). The dissimilarity between susceptible (‘BRS Pampa CL’ = Fig. 3g) and resistant (‘Dawn’ = Fig. 3c,d) cultivars to RWW attack in terms of density and lignification of sclerenchyma cells was confirmed by the histochemical method, which revealed the reddish color of lignified structures (Fig. 3c,d,g).

#### Enzyme assays and biochemical estimations

**Figure 4:**
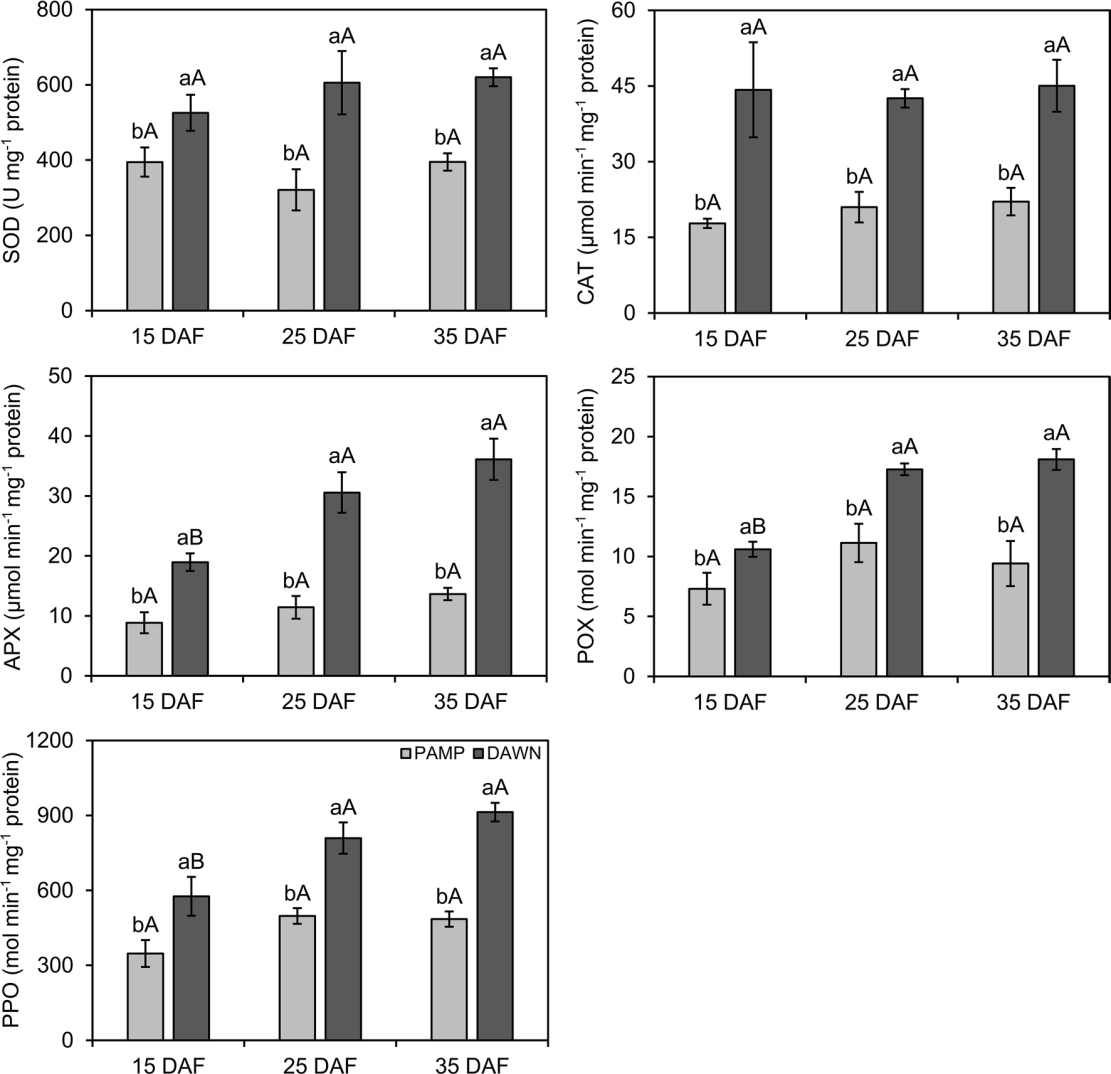
Activities (± SE) of superoxide dismutase (SOD), catalase (CAT), ascorbate peroxidase (APX), peroxidase (POX), and polyphenol oxidase (PPO) in rice roots from susceptible (Ss) and resistant (Rr) cultivars with natural infestations of the South American rice water weevil *Oryzophagus oryzae* at 15, 25, and 35 days after flooding (DAF). Rice cultivars: PAMP = BRS Pampa CL (Ss) (light gray columns); DAWN = Dawn (Rr) (dark gray columns). Sampling times corresponded to beginning (15 DAF), peak (25 DAF), and decline (35 DAF) phases of larvae infestations in the field. Average numbers of larvae on plants collected for biochemical analysis at 15 DAF: PAMP = 4.94; DAWN = 4.15; 25 DAF: PAMP = 16.56, DAWN = 4.33; 35 DAF: PAMP = 20.81, DAWN = 5.08. Different lowercase letters above bars denote significant differences among rice cultivars at each sampling time, whereas different uppercase letters above bars denote significant differences in the same rice cultivar among sampling times, both by the Scott Knott test (*P* ≤ 0.05).

**Figure 5:**
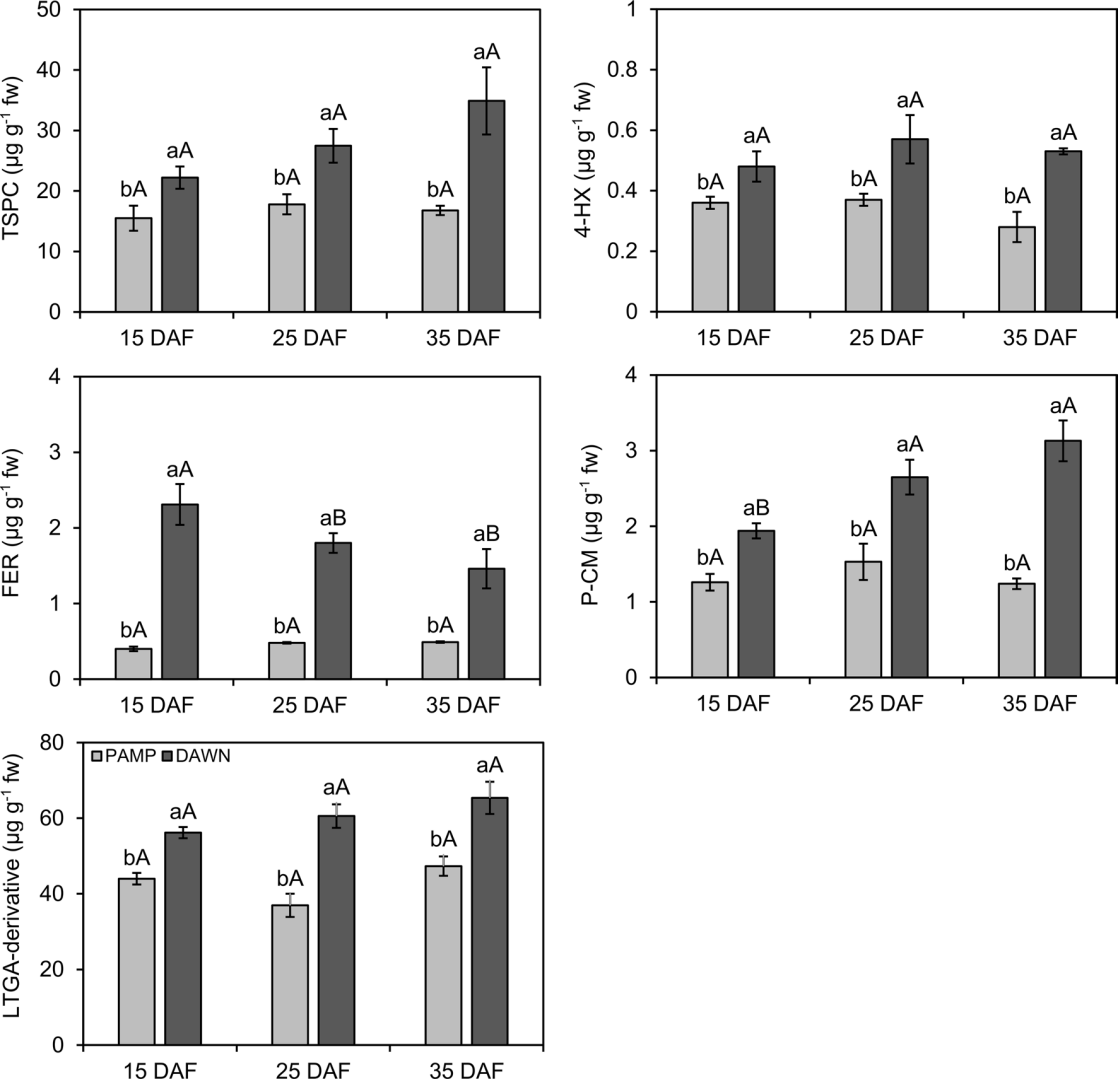
Concentrations (± SE) of total soluble phenolic compounds (TSPC), 4-hydroxybenzoic (4-HX), ferulic (FER), and *p*-coumaric (P-CM) phenolic acids, and lignin-thioglycolic acid (LTGA) derivatives in rice roots from susceptible (Ss) and resistant (Rr) cultivars with natural infestations of the South American rice water weevil *Oryzophagus oryzae* at 15, 25, and 35 days after flooding (DAF). Rice cultivars: PAMP = BRS Pampa CL (Ss) (light gray columns); DAWN = Dawn (Rr) (dark gray columns). Sampling times corresponded to beginning (15 DAF), peak (25 DAF), and decline (35 DAF) phases of larvae infestation in the field. Average larvae number on plants collected for biochemical analysis at 15 DAF: PAMP = 4.94; DAWN = 4.15; 25 DAF: PAMP = 16.56, DAWN = 4.33; 35 DAF: PAMP = 20.81, DAWN = 5.08. Different lowercase letters above bars denote significant differences among rice cultivars at each sampling time, whereas different uppercase letters above bars denote significant differences in the same rice cultivar among sampling time, both by the Scott Knott test (*P* ≤ 0.05).

Significant differences were found in enzyme activities (SOD: df = 1, F = 27.41, *P* ≤ 0.001; CAT: df = 1, F = 53.17, *P* ≤ 0.001; APX: df = 1, F = 102.98, *P* ≤ 0.001; POX: df = 1, F = 49.65, *P* ≤ 0.001; PPO: df = 1, F = 57.99, *P* ≤ 0.001) and concentrations of TSPC (df = 1, F = 32.84, *P* ≤ 0.001) and LTGA (df = 1, F = 24.43, *P* ≤ 0.001) between cultivars at 15, 25, and 35 DAF, but without significant interactions between ‘cultivar’ and ‘sampling time’ (SOD: df = 2, F = 1.20, *P* = 0.31; CAT: df = 2, F = 0.93, *P* ≤ 0.001; APX: df = 2, F = 0.32, *P* = 0.73; POX: df = 2, F = 2.42, *P* = 0.11; PPO: df = 2, F = 1.85, *P* = 0.17; TSPC: df = 2, F = 1.10, *P* = 0.35; LTGA: df = 2, F = 0.87, *P* = 0.43). Enzyme activities and concentrations of TSPC and LTGA were higher in ‘Dawn’ than in ‘BRS Pampa CL’, regardless of the sampling time (Figs. 4, 5). Biochemical estimations remained similar for each cultivar among sampling times, except for APX, POX, and PPO in ‘Dawn’, which showed less activity at 15 than at 25 and 35 DAF (Figs. 4, 5).

**Figure 6:**
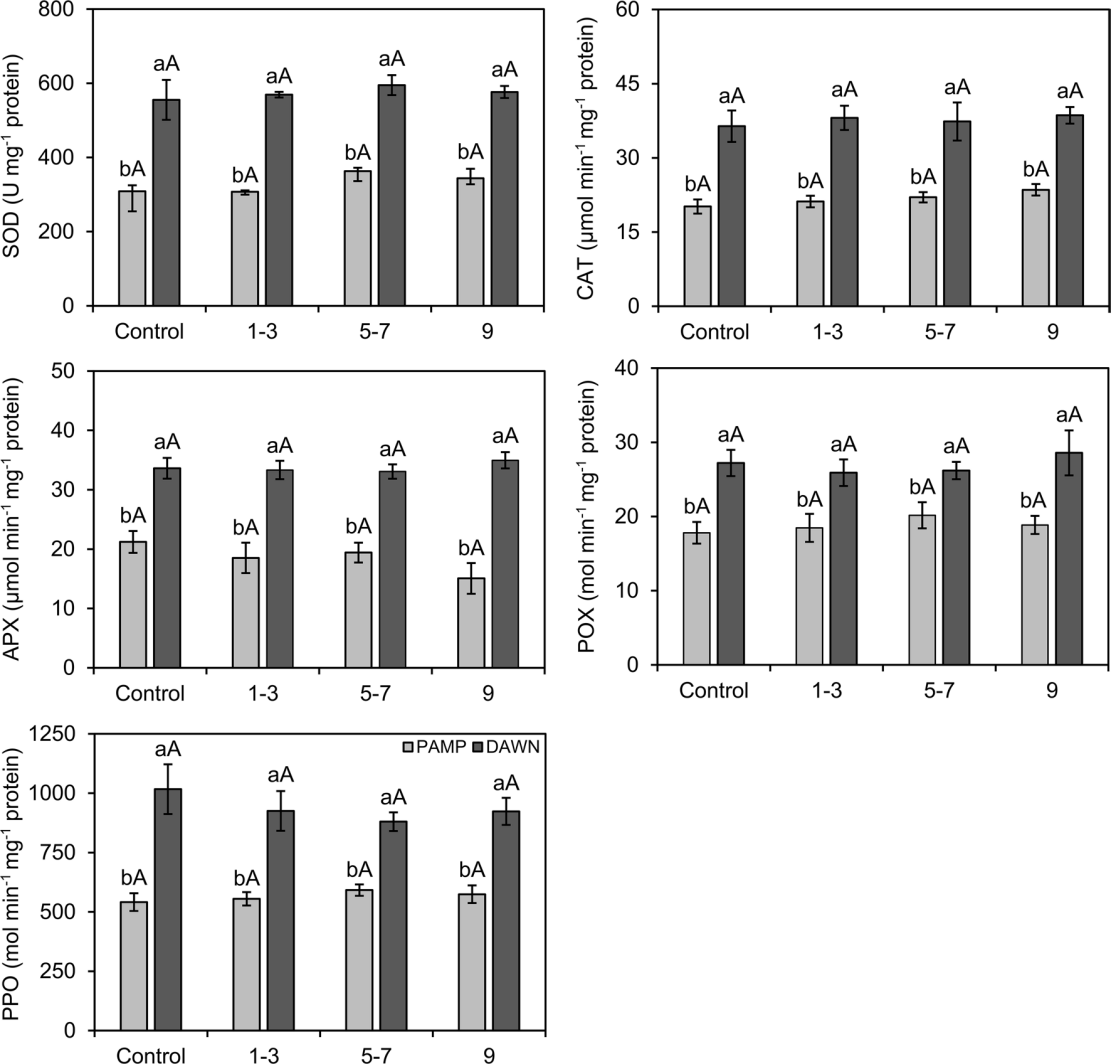
Activities (± SE) of superoxide dismutase (SOD), catalase (CAT), ascorbate peroxidase (APX), peroxidase (POX), and polyphenol oxidase (PPO) in rice root from susceptible (Ss) and resistant (Rr) cultivars with different levels of natural infestation of the South American rice water weevil *Oryzophagus oryzae* at 25 Days After Flooding (DAF). Rice cultivars: PAMP = BRS Pampa CL (Ss) (light gray columns); DAWN = Dawn (Rr) (dark gray columns). Levels of natural infestation of larvae on plants selected for biochemical analysis at 25 DAF (larvae infestation peak in the field) = 1 (control): 0 larva; 2: 1–3 larvae; 3: 5–7 larvae; 4: 9 larvae. Different lowercase letters above bars denote significant differences among rice cultivars at each infestation level, whereas different uppercase letters above bars denote significant differences in the same rice cultivar among infestation level, both by the Scott Knott test (*P* ≤ 0.05).

**Figure 7:**
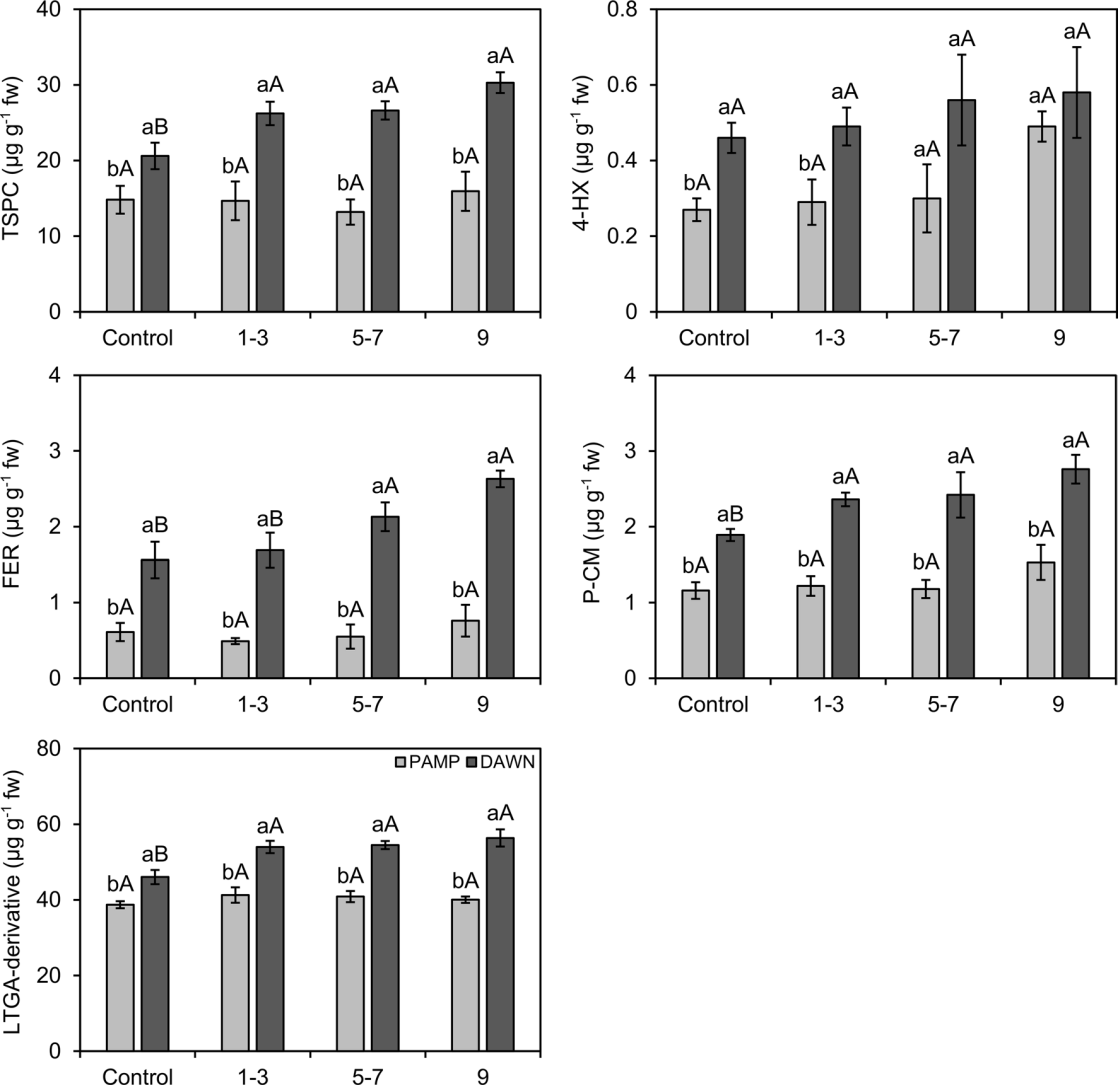
Concentrations (± SE) of total soluble phenolic compounds (TSPC), 4-hydroxybenzoic (4-HX), ferulic (FER), and *p*-coumaric (P-CM) phenolic acids, and lignin-thioglycolic acid (LTGA) derivatives in rice roots from susceptible (Ss) and resistant (Rr) cultivars with different levels of natural infestation of the South American rice water weevil *Oryzophagus oryzae* at 25 days after flooding (DAF). Rice cultivars: PAMP = BRS Pampa CL (Ss) (light gray columns); DAWN = Dawn (Rr) (dark gray columns). Levels of natural infestation of larvae on plants selected for biochemical analysis at 25 DAF (larvae infestation peak in the field) = 1 (control): 0 larva; 2: 1–3 larvae; 3: 5–7 larvae; 4:9 larvae. Different lowercase letters above bars denote significant differences among rice cultivars at each infestation level, whereas different uppercase letters above bars denote significant differences in the same rice cultivar among infestation level, both by the Scott Knott test (*P* ≤ 0.05).

Under progressive levels of natural infestations of larvae in the plots at 25 DAF (peak of larval incidence in the field), enzyme activities and concentrations of TSPC and LTGA were again higher in ‘Dawn’ (SOD: df = 1, F = 215.60, *P* ≤ 0.001; CAT: df = 1, F = 121.57, *P* ≤ 0.001; APX: df = 1, F = 120.89, *P* ≤ 0.001; POX: df = 1, F = 38.28, *P* ≤ 0.001; PPO: df = 1, F = 102.31, *P* ≤ 0.001; TSPC: df = 1, F = 77.21, *P* ≤ 0.001; LTGA: df = 1, F = 79.24, *P* ≤ 0.001), but without significant interactions between the “cultivar” and “infestation” factors (SOD: df = 3, F = 0.91, *P* = 0.45; CAT: df = 3, F = 0.28, *P* = 0.84; APX: df = 3, F = 0.71, *P* = 0.55; POX: df = 3, F = 0.68, *P* = 0.57; PPO: df = 3, F = 0.88, *P* = 0.46; TSPC: df = 3, F = 1.67, *P* = 0.10; LTGA: df = 3, F = 2.35, *P* = 0.06) (Figs. 5, 6). There were no significant differences in SOD, CAT, APX, POX, and PPO activities among infestation levels (2 = 1–3 larvae; 3 = 5–7 larvae; 4 = 9 larvae) and non-infested plants (1 = 0 larva), except for TSPC and LTGA in ‘Dawn’, in which concentrations in non-infested roots were significantly lower than in infested roots at different levels of larval infestation (Figs. 6, 7).

The phenolic acids 4-hydroxybenzoic (4-HX), ferulic (FER), and *p*-coumaric (P-CM) were identified from the roots of both cultivars (Figs. 5, 7). Significant differences were observed in concentrations of 4-HX, FER, and P-CM between cultivars at 15, 25, and 35 DAF (4-HX: df = 1, F = 22.60, *P* ≤ 0.001; FER: df = 1, F = 630.63, *P* ≤ 0.001; P-CM: df = 1, F = 69.72, *P* ≤ 0.001). There were also significant interactions between “cultivar” and “sampling time” for FER and P-CM (FER: df = 2, F = 11.86, *P* ≤ 0.001; P-CM: df = 2, F = 7.12, *P* = 0.005). Higher concentrations of the phenolic acids 4-HX, FER, and P-CM were detected in ‘Dawn’ at all sampling times (Fig. 5). ‘Dawn’ displayed an increase in P-CM and a decrease in FER over the sampling times, e.g., P-CM and FER concentrations were highest at 35 and 15 DAF, respectively. The concentrations of 4-HX remained unaltered across the sampling times (Fig. 5).

Significant differences were observed in the concentrations of 4-HX, FER, and P-CM between cultivars under progressive levels of larval infestation (4-HX: df = 1, F = 12.25, *P* = 0.003; FER: df = 1, F = 82.69, *P* ≤ 0.001; P-CM: df = 1, F = 83.31, *P* ≤ 0.001). The concentrations of phenolic acids were again higher in ‘Dawn’ (Fig. 7). In addition, there were significant differences in the concentrations of FER and P-CM with respect to the “infestation” factor (FER: df = 3, F = 3.56, *P* = 0.04; P-CM: df = 3, F = 4.52, *P* = 0.02). FER and P-CM concentrations were significantly higher in infested (2 = 1–3 larvae; 3 = 5–7 larvae; 4 = 9 larvae) than in non-infested ‘Dawn’ plants (1 = 0 larva). The highest concentration of FER in ‘Dawn’ was observed in infestation levels 3 (5–7 larvae) and 4 (9 larvae), which differed from concentrations in infestation levels 1 (0 larva) and 2 (1–3 larvae) (Fig. 7). The concentrations of 4-HX, which were different between cultivars, were not different between non-infested and infested plants (4-HX: df = 3, F = 2.04, *P* = 0.15) (Fig. 7).

**Figure 8:**
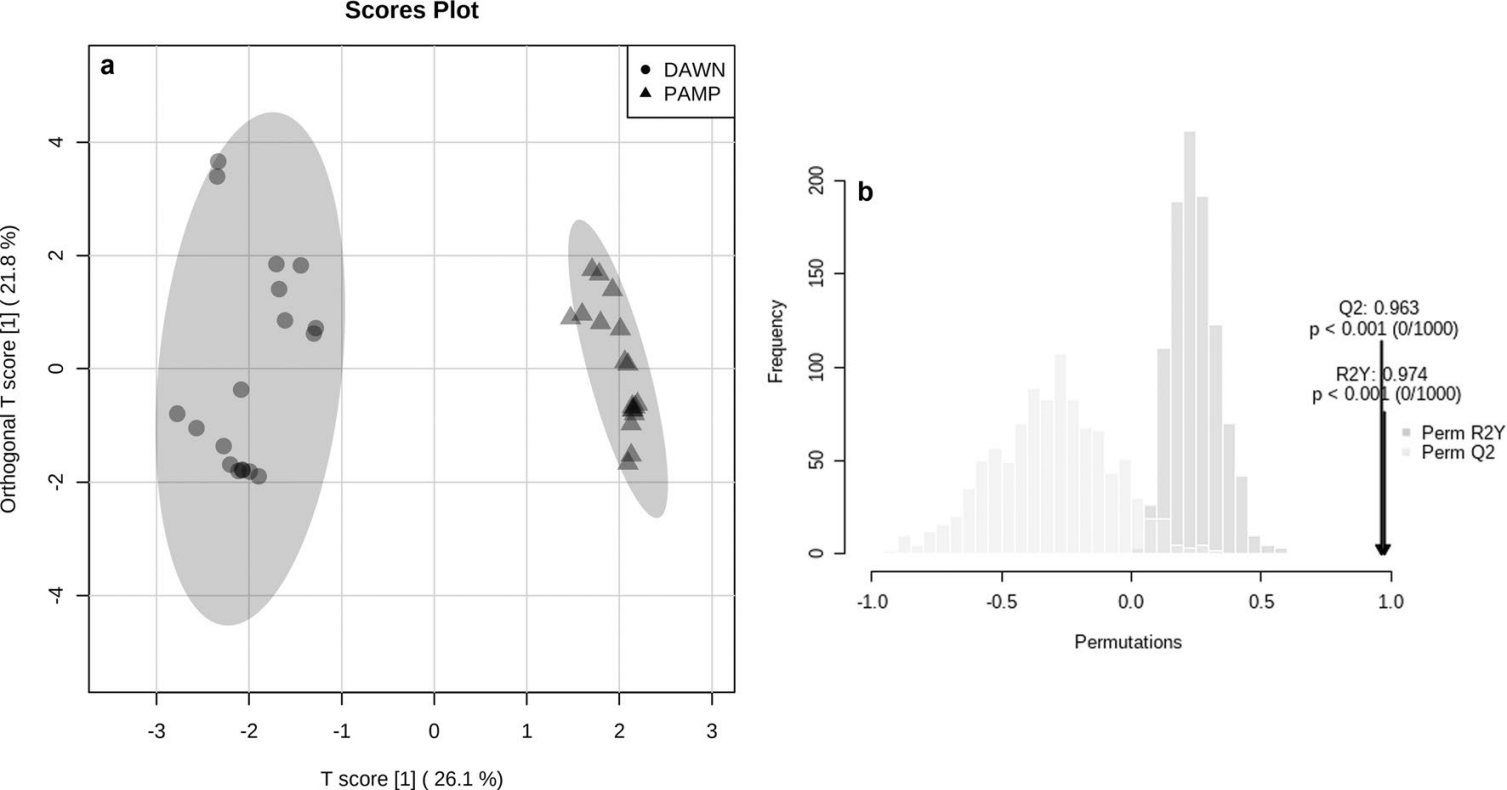
Score plots of orthogonal projection to latent structure discriminant analysis (OPLS-DA) (**a**) and model validation by the Permutation test (1000 permutations) (**b**) derived from LC–MS/MS dataset of two rice cultivars of contrasting susceptibility to the South American rice water weevil *Oryzophagus oryzae* (Resistant: DAWN = ‘Dawn’; Susceptible: PAMP = ‘BRS Pampa CL’). The *P* value based on permutation was *P* < 0.001 (0/1000).

In the untargeted metabolomics approach, OPLS-DA was applied to investigate metabolites differentially accumulated in ‘Dawn’ and ‘BRS Pampa CL’. The score plot of the OPLS-DA model exhibited a complete separation among root samples from ‘Dawn’ and ‘BRS Pampa CL’ (Fig. 8a). The robustness of the model was tested using a random Permutation test with 1000 permutations (Fig. 8b). This test provided an R^2^Y of 0.97 (*P* ≤ 0.001), Q^2^ of 0.96 (*P* ≤ 0.001), and a difference between R^2^Y and Q^2^ of 0.01 (< 0.30), indicating that the model was not overfitted and had a good predictive ability to distinguish the rice cultivars under study. Metabolites that accumulate to a significant degree and that distinguished ‘Dawn’ from ‘BRS Pampa CL’ were identified based on the Student’s t-test (FDR-adjusted *P* ≤ 0.05) and FC of ≥ 2.0. The Volcano plots highlight metabolites with FDR-adjusted *P* ≤ 0.05 and FC of ≥ 2.0 (Supplementary Fig. S1). In total, three compounds accumulated differentially between cultivars. The contents of annotated compounds 1-*p*-coumaroyl-3-feruloylglycerol (FC of 4.66), corchorifatty acid F (3.85), and *p*-Coumaric acid ethyl ester (3.69) were significantly higher in the ‘Dawn’ group compared to the ‘BRS Pampa CL’ group, with FDR-adjusted *P* < 0.001 (Table 2).

**Table 2:** Metabolites detected by LC–MS/MS analyses whose content was differentially accumulated between South American rice water weevil (*Oryzophagus oryzae*)-resistant and susceptible rice cultivars [*P* ≤ 0.05 and Fold Change (FC) ≥ 2.0].

Annotation	RT (min)	*m/z*		Error (ppm)	mSigma	Fragments	Database	Adducts	FC*	FDR (*P*)**
		Experimental	Theoretical							
1-*p*-Coumaroyl-3-feruloylglycerol	13.30	413.1247	413.1242	− 2.6	39.4	413.1253 (100.0); 193.0502 (82.5); 163.0383 (76.7)	Sarvestani (2016)	M-H	4.6632	6.2867E−8
Corchorifatty acid F	13.05	327.2171	327.2177	1.8	60.9	327.2160 (100.0); 211.1346 (33.0); 229.1443 (23.4)	FDB014713	M-H	3.8520	4.1205E−8
*p*-Coumaric acid ethyl ester	11.13	237.0768	237.0768	0.2	31.5	145.0296 (100.0); 119.0502 (39.6); 117.0344 (32.6); 163.0405 (15.1)	FDB000258	M + FA-H	3.6921	6.4972E−8

*A positive FC-value means the feature was increased in the resistant (‘Dawn’) relative to the susceptible (‘BRS Pampa CL’) group.

**FDR-adjusted *P* value according to Student’s t test.

## Discussion

Plant resistance is a key component of the integrated management programs for RWWs and for sustainable rice production worldwide. From this perspective, the present study investigated the factors potentially involved in rice resistance to *O. oryzae*. Understanding the mechanisms involved in rice plant-RWW interactions, which are not yet completely characterized^21^, is essential for the successful development of elite cultivars that are resistant to insects, since the degree of resistance of a cultivar is related to the effectiveness of plant defensive traits against herbivory^24,27^.

Based on preferences for adult feeding and oviposition, a lack of antixenosis was found among the evaluated cultivars, including ‘Dawn’, which is known to be resistant to RWW^19,31^. The results obtained herein showed that there was no interference in host selection by the evaluated cultivars, since all the cultivars were equally chosen by RWWs for feeding and oviposition.

The expression of antixenosis often results from morphological or chemical traits in plants that negatively affect the natural behavior of arthropods during the process of host selection, with antixenotic plants less frequently used by herbivores for food, oviposition, or shelter in relation to other plants under the same infestation conditions^22^. Morphoanatomical factors in rice plants, such as plant height, density of trichomes on leaves, epidermal tissue thickness, number and length of internodes, and internal and external diameters of stems have been directly associated with antixenosis against species of stem borers^25,35–38^. Antixenosis in different rice genotypes has been reported to affect the feeding and oviposition of RWW adults^17,18,20^. Among these studies, Saad et al.^20^ measured the variation among cultivars in plant height and intraveinal distance to explain variation in ovipositional preference by *L. oryzophilus* female, asnd none of the measured traits were related to ovipositional preference. According to Moreira^39^, plants with a higher number of tillers provide more favorable oviposition sites for *O. oryzae*; however, this finding was not confirmed in a recent study by Pazini et al.^40^. Our research was the first to conduct an in-depth study of morphoanatomical traits associated with rice resistance to the RWW. In the present study, the lack of differences observed among the cultivars in anatomical and morphological characteristics of leaves is consistent with, and may explain, the lack of antixenosis in the cultivars ‘BRS Pampa CL’ and ‘Dawn’ during the host selection process of *O. oryzae*.

Although equally selected, with quantitatively similar feeding and oviposition rates, ‘BRS Pampa CL’ differed from ‘Dawn’ in terms of indicators of antibiosis (Table 1). ‘BRS Pampa CL’ harbored larvae in greater numbers and with greater body weight. The time required for 50% emergence of adults from ‘BRS Pampa CL’ was almost two times shorter than the time required for 50% emergence from ‘Dawn’; in addition, adults originating from ‘BRS Pampa CL’ had a higher body weight than those from ‘Dawn’ (Table 1). Antibiosis effects have already been demonstrated to affect the larval growth of *L. oryzophilus* in cultivars Nira and Jefferson^17,20^, and larval survival and adult emergence of *O. oryzae* were shown to be reduced in ‘Dawn’^19^ and ‘BR IRGA 417’, respectively^41^.

The equal suitability of ‘Dawn’ and ‘BRS Pampa CL’ for adult feeding and preference was not correlated with the performance of progeny on roots of ‘Dawn’. Because host plants have crucial effects on herbivore population dynamics, Stenberg and Muola^42^ pointed out that adult individuals often colonize and remain on plants that are most palatable to their offspring. When considering that *O. oryzae* and other RWWs are specialized on aquatic grasses, such as plants in the genus *Oryza*^43^, it is expected that the host plant has developed some defensive and survival strategies against herbivory. In the present study, characterization of potential resistance-related traits represents an advance in existing knowledge because it supports the hypothesis of the presence of direct defense factor(s) in roots that mediate larval and adult antibiosis.

Antioxidant enzymes, either constitutive or inducible, have been associated with the resistance of host plants against insects and pathogens^44^. In the present study, as early as the beginning of the natural infestation of *O. oryzae* in the rice field at 15 DAF, the activities of the enzymes SOD, CAT, APX, POX, and PPO were higher in ‘Dawn’. They remained high and were consistently higher in ‘Dawn’ than in ‘BRS Pampa CL’ during the other key periods of population fluctuation of the pest, at 25 DAF and 35 DAF. Increases of approximately 90, 71 and 58% in the activities of APX, POX and PPO, respectively, were observed from 15 to 35 DAF in ‘Dawn’. Enzymatic activities were also higher in the cultivar Dawn than the cultivar BRS Pampa CL in plants under progressive larval stress at the peak of *O. oryzae* infestation in the rice field (25 DAF).

The higher activities of antioxidant enzymes in both infested and non-infested ‘Dawn’ plants at different periods of infestation suggest that the antioxidant system in ‘Dawn’ roots was constitutively more efficient. Han et al.^44^ detected high constitutive activities of the enzymes phenylalanine ammonia lyase (PAL), POX, and PPO in wheat cultivars with resistance to *Sitobion avenae* (Fabricius) (Hemiptera: Aphididae), which were quantitatively higher than in susceptible cultivars at different stages of the phenological cycle of wheat plants. Constitutive activity of POX and PPO in a fava bean cultivar resistant to *Aphis craccivora* Koch (Hemiptera: Aphididae) was reported in the study by Soffan et al.^26^, and there were no significant differences in enzymatic activities in plants that were infested and not infested with aphids.

In the comparison of infested and non-infested plants at 25 DAF made here, it is important to consider that antioxidant enzymes have been associated wth signal transduction and rapid activation of plant defense signaling^45^. Jesus et al.^46^ found no significant differences in POX activity between infested and non-infested plants at 5, 10, and 15 days after infestation of a soybean genotype expressing antibiosis against *Aphis glycines* Matsumura (Hemiptera: Aphididae). In this context, the reaction of ‘Dawn’ to larval feeding may have been rapid, and it is not possible to rule out the occurrence of peak enzymatic activities before 25 DAF, as during the endophytic oviposition activity in the host selection process^47^. In rice, the enzymes chitinase (CHI), PAL, POX, and PPO showed greater activities in resistant than in susceptible cultivars in response to herbivory with different pests, such as *Cnaphalocrosis medinalis* (Guenée) (Lepidoptera: Pyralidae), *Scirpophaga incertulas* (Walker) (Lepidoptera: Pyralidae), *Nilaparvata lugens* (Stal) (Hemiptera: Delphacidae) and *Laodelphax striatellus* Fallén (Hemiptera: Delphacidae)^27,28^.

In this study, the concentrations of the hydroxycinnamic acid derivatives (FER, P-CM, and annotated compounds 1-*p*-coumaroyl-3-feruloylglycerol and *p*-Coumaric acid ethyl ester), hydroxybenzoic acid derivatives (4-HX), and an annotated compound belonging to the family of linoleic acid derivatives (Corchorifatty acid F) were significantly higher in ‘Dawn’. Corchorifatty acid F has been isolated from rice infected with *Pyricularia oryzae* Cavara, causal agent of blast disease, and seemed to be active against the pathogen in resistant rice cultivars^48^. The presence of this annotated compound in the cultivar Dawn is consistent with the fact that ‘Dawn’ is resistant to most of the races of *P. oryzae*^32,49^.

Phenolic compounds play an important role in antibiosis against insects because of direct anti-nutritional or cytotoxic effects and their role in strengthening plant cell walls. Incorporation of the phenolic acids FER and P-CM in a diet, simulating concentrations found in resistant maize leaves, caused antibiosis effects on the growth and development of *Chilo partellus* (Swinehoe) (Lepidoptera: Pyralidae)^50^. Some studies have shown the participation of phenolic metabolites, such as some hydroxybenzoic and hydroxycinnamic acids (mainly P-CM and FER and their esters), in the strengthening and lignification of the plant cell wall^51–54^. An evaluation of incorporation of phenolic acids into monocot lignins was performed by Sarvestani^55^ and the most abundant compound in corn plants found was 1-*p*-coumaroyl-3-feruloylglycerol, a compound that is also present at lower levels in other grasses. Compounds of this kind act directly in the association between hemicellulose and lignin in cell walls, strengthening then and leading to difficulties in chewing/penetrating and ingesting plant tissues, thus reducing the quantity and quality of nutrients and causing direct cytotoxicity in insects^56,57^. Ralph and Landucci^58^ and Shivashankar et al.^59^ reported that high concentrations of lignin and P-CM lead to the synthesis of lignin related to plant defense. The higher concentration of LTGA in the root of ‘Dawn’ has also been histochemically demonstrated at levels that are visibly different from ‘BRS Pampa CL’ (Fig. 3c,d,g). It is important to note that the lignin-reinforced sclerenchyma in ‘Dawn’ was also visible in roots lacking larval infestation (Fig. 3d) at 25 DAF. Thus, this characteristic [not clear what characteristic you are referring to] may be, in part, constitutively associated with this cultivar. A similar diagnosis in roots of rice genotypes with resistance to *Meloidogyne graminicola* Golden and Birchfield (Nematoda: Meloidogynidae) was developed by Galeng-Lawilao et al.^60^.

Phenols may also be oxidized by the action of PPO and some peroxidases (POX, APX, and others), in the presence of O_2_ or H_2_O_2_, leading to the formation of reactive *o*-quinones^24^. Quinones bind to proteins and carbohydrates in plant tissue, reducing the availability and biological value of proteins, and they may also exhibit toxicity to insects^61^. The occurrence of disorders in insect nutrition, caused by anti-nutritional factors in host plants, comprises one of the most important aspects of plant resistance to insects^24^. Rigsby et al.^62^ found that the high enzymatic activity of POX, followed by lignin polymerization and quinone generation, in *Fraxinus* spp., decreased the nutritional quality of the host and served as a determinant for resistance to the emerald ash borer *Agrilus planipennis* Fairmaire (Coleoptera: Buprestidae). Larval growth and adult emergence of *Bactrocera cucurbitae* (Coquillett) (Diptera: Tephritidae) are strongly inhibited in Curcubitaceae (*Sechium edule*), which exhibits higher enzyme activities [tyrosine ammonia-lyase (TAL), cinnamyl-alcohol dehydrogenase (CAD), PAL and POX] and phenolic acids (P-CM) involved in lignin biosynthesis^59^. Our results showed that resistance in ‘Dawn’ may have resulted from such effects. ‘Dawn’ and ‘BRS Pampa CL’, which were similar in terms of preference for feeding and oviposition, supported markedly different larval densities. The surviving larvae in ‘Dawn’ also showed marked malnutrition, as evidence by lower weights, possibly due to the higher activities of enzymes such as POX and PPO and the abundant presence of phenolic metabolites and lignin in the roots of ‘Dawn’, detected during the critical periods of larval occurrence in the field. Transgenerational effects, which prolonged the time of emergence and reduced the body weight of adult offspring, were observed because of larval antibiosis (Table 1).

The information obtained in this study represents a fundamental and novel step toward elucidating the factors of rice plants associated with resistance to *O. oryzae*. Knowledge of this kind is essential since it can be used to develop biochemical or morphological markers for resistance to RWWs^13^. Thus, potentially resistant materials could be selected early in the development process of a cultivar, avoiding sampling of the larval population in extensive screenings, which are very difficult to perform in the field. The findings of the present study allow us to infer the potential use of lignin and oxidoreductive enzymes (POX and PPO) in roots as morphological and biochemical markers, respectively, for identifying rice genotypes with antibiosis to *O. oryzae* larvae in rice breeding programs. Despite the encouraging prospects, it will be necessary to validate whether rice genotypes resistant to other species of RWWs from other rice-producing regions worldwide exhibit the same resistance-related traits. In this sense, standard methods under controlled conditions and no-choice experiments can be developed to validate resistance. In addition, further studies are needed to confirm the constitutive or induced nature of defense factors and identify genes controlling resistance mechanisms to develop new rice populations by techniques involving genetic manipulation and molecular breeding (e.g., gene mapping, gene cloning, and selection of lines with resistance to RWW).

## Methods

### Field screenings for host plant resistance to RWWs

Evaluations of rice cultivars for resistance to *O. oryzae* were conducted over two consecutive years (2016/17 and 2017/18), in leveled plots of a typic Albaqualf soil on the edge of a 100-hectare area at the Embrapa Temperate Agriculture Research Station, located near Pelotas, RS, Brazil (31° 48′ 45″ S, 52° 27′ 59″ W). This site historically experiences high *O. oryzae* infestations and has been used in studies of resistance of rice to RWW^19,21,41^.

Six commercial rice cultivars were selected as treatments in the 2-year choice field experiment (Supplementary Table S1). The experimental design was a Latin-square (six treatments and six replications) to provide an equal probability of natural RWW infestations in all treatments^21^. Each plot measured 4.56 m^2^ with five rows of rice spaced 30 cm apart, where each row had 20 plants spaced 20 cm apart (100 plants total). Each plot was separated from neighboring plots by 1 m on all sides. Seedlings produced in a greenhouse were transplanted into plots 20 days after emergence, in October. Three days after transplanting seedlings, the flood was gradually increased to a depth of approximately 15 cm, which is the preferred condition for the natural infestation of *O. oryzae*^63^. The plots were kept flooded until they were drained for harvest. Rice plots were not treated with any pesticides throughout the growing season to allow for natural infestations of RWWs. Weed control was carried out by hand weeding.

#### RWW feeding and oviposition behavior

Feeding preference of RWW on cultivars was determined by counting densities of feeding scars on leaves of 30 plants randomly chosen from each plot at 5, 8, and 11 days after the permanent flooding (DAF = days after flooding). At these same sampling times, six plants were sampled from each plot and egg densities were quantified in leaf-sheaths. For this, leaf-sheaths were kept in 75% alcohol for bleaching and counted using the bottom light of a stereomicroscope (Olympus SZ, Olympus Corporation, Tokio, Japan) according to the procedures used by Lanka et al.^64^.

#### RWW density and performance

Population densities of immature RWW (larvae and pupae) in plots were determined following the collection method of Neves et al.^41^ at 15, 25, and 35 DAF, representing the beginning, peak, and decline phases of larval populations in the field, respectively^65^. Six samples with one plant each were collected from each plot at each sampling time using a soil-root core sampler with diameter of 10 cm and depth of 9 cm. Larvae dislodged from each root samples during sampling procedures in each plot were counted and transferred to 2.9 × 11.5 cm polypropylene tubes containing approximately 40 mL water. The tubes were kept in a climate-controlled room (25 ± 2 °C) for up to two days to record the individual larval weight following the method of Lima et al.^21^.

The effect of rice cultivars on production of F1-generation adult offspring of larvae was determined according to Lima et al.^21^. Four additional core samples were collected from each plot at 30 DAF and transferred into 30 × 50-cm plastic buckets (30 L) containing ~ 7 L water. In the greenhouse, the samples from each plot were grouped and submerged in water in buckets covered by voile tissue to capture and count adults emerging from roots. Counting of offspring adults began four days after placement of core samples in the buckets, continuing at four-day intervals until no adult was found for three consecutive sampling dates. The time (days) required to reach 50% adult population emergence was calculated for each cultivar (TE50%). Additionally, adults captured from the buckets on the assessment days were kept in the laboratory (8 ± 2 °C) for up to two days in plastic microtubes (2 mL) for individual weighing (mg).

### Defense-associated traits in rice cultivars with contrasting susceptibility

Resistance-associated traits to *O. oryzae* were determined in two cultivars of contrasting susceptibility to attack, which were selected based on field screenings, as described above.

#### Morphoanatomical traits

For leaf tissue traits, samples 5 cm in length were excised from the central region of the youngest fully expanded leaf of the main stem from the rice plants used for the feeding and oviposition preference assessment at 8 DAF. For root tissue traits, samples from the first 9 cm of the root system were excised from the plants used to determine larval densities at 25 DAF. The first 9 cm of rice plant roots was selected because it harbors the highest larval infestation in the field^41^. Both leaf and root tissue traits were observed using samples from one plant from each of the six plots per cultivar. Immediately after sampling, the leaf and root pieces were fixed in Karnovsky’s solution^66^ and dehydrated in a graded ethanol series.

For morphological-surface characterization, leaf tissue samples of both cultivars were treated with liquid carbon dioxide in a critical point dryer (EM CPD 300, Leica Microsystems, Wetzlar, Germany). Dried samples were mounted on a metal stub and coated with gold using a sputter‐coater (Desk V, Denton Vacuum, Moorestown, USA). The fine-detail morphological characters (adaxial and abaxial surfaces of leaf tissue) were examined and photographed using a scanning electron microscope (JEOL JSM-6610, JEOL, Tokyo, Japan), operated at an accelerating voltage of 15 kV and 15–17 mm working distance.

For anatomical characterization, leaf and root tissue samples were embedded in plastic resin (Leica HistoResin, Leica Biosystems, Wetzlar, Germany) according to manufacturer’s instructions. Seven-micron-thick leaf and root tissue cross-sections were cut using a rotary microtome (ANCAP 297, ANCAP Equipamentos eletro-eletrônicos, São Paulo, Brazil). The cross-sections were stained with 0.05% toluidine blue O in citrate–phosphate buffer (pH = 4.5) and mounted in Entellan synthetic resin (Merck, Darmstadt, Germany). In addition, histochemical tests for detection of root-structural phenolic compound (lignin) were performed. In this procedure, the root cross-sections were stained with 2% phloroglucinol-HCl^67^. Leaf and root morphoanatomical traits of both cultivars were observed by images digitally captured with a Leica DC 300F camera (Leica Microsystems, Wetzlar, Germany) coupled with a Discovery V20 microscope (Zeiss, Göttingen, Germany).

#### Enzyme assays and biochemical estimations

Biochemical analyses were performed using the first 9 cm of the root system^41^ from plants used for larval sampling at 15, 25, and 35 DAF. Immediately after sampling, the first 9 cm of roots were frozen in liquid nitrogen and stored at −80 °C.

The crude extract for enzyme assays was obtained according to Dorneles et al.^68^. The activities of superoxide dismutase (SOD, EC 1.15.1.1), catalase (CAT, EC 1.11.1.6), peroxidase (POX, EC 1.11.1.7), and polyphenoloxidase (PPO, EC 1.10.3.1) were determined according to Dorneles et al.^68^; the activity of ascorbate peroxidase (APX, EC 1.11.1.11) was determined according to Nakano and Asada^69^. SOD activity was quantified based on the colorimetric quantification of nitroblue tetrazolium (NBT) (Sigma-Aldrich, São Paulo, Brazil) photoreduction; the specific enzymatic activity was expressed in units of SOD mg^-1^ of protein based on the amount of the enzyme that inhibited NBT photoreduction by 50%. CAT activity was quantified using hydrogen peroxide (H_2_O_2_) as substrate (Merck, São Paulo, Brazil) and expressed as micromoles of H_2_O_2_ degraded min^−1^ mg^−1^ of protein. POX and PPO activities were quantified based on the colorimetric quantification of pyrogallol (Sigma-Aldrich, São Paulo, Brazil) oxidation and expressed as moles of purpurogallin min^−1^ mg^−1^ of protein using an extinction coefficient of 2.47 mM cm^−1^. APX activity was determined based on the quantification of the ascorbate oxidation rate (Sigma-Aldrich, São Paulo, Brazil) and expressed as μmol of oxidized ascorbate min^−1^ mg^−1^ of protein. Total protein content was obtained using the Bradford method, with bovine serum albumin as a standard^70^.

Total soluble phenolic compounds (TSPC) and lignin and lignin-like phenolic polymers were extracted from 0.1 g of root samples as described by Dallagnol et al.^71^. TSPC content was estimated using Folin-Ciocalteu reagent (Sigma-Aldrich, São Paulo, Brazil). A standard calibration curve was prepared using pyrogallic acid (Synth, Diadema, Brazil), and the content of TSPC was expressed as μg of pyrogallic acid equivalents per g of fresh matter (fm). Dried alcohol-insoluble residue from the TSPC extraction and thioglycolic acid (Sigma-Aldrich, São Paulo, Brazil) were used to determine the lignin content [lignin-thioglycolic acid (LTGA) derivatives]^68^. LTGA was quantified based on colorimetry and expressed as μg g^−1^ of fm using alkali lignin, 2-hydroxypropyl ether (Sigma-Aldrich, São Paulo, Brazil) as the standard. SOD, CAT, POX, PPO, APX, protein, TSPC, and LTGA quantifications were performed using a spectrophotometer Bel UV-UM51 (Bel Engineering, Milan, Italy).

The same extract used for TSPC quantification was assayed by liquid chromatography-mass spectrometry (LC–MS/MS) using a targeted approach for the analysis of 4-hydroxybenzoic acid, ferulic acid, and *p*-coumaric acid (other targeted phenolics—caffeic acid, chlorogenic acid, gallic acid, protocatechuic acid, synaptic acid, syringic acid, and vanillic acid—were either not present or below detection limits) and a non-targeted metabolomics approach. LC–MS/MS analysis was performed on a high-performance liquid chromatography system (UFLC, Shimadzu, Kyoto, Japan) coupled to a quadrupole time-of-flight mass spectrometry detector (Q-TOF-MSD) (Impact HD, Bruker Daltonics, Bremen, Germany).

Separation of compounds was performed using a C18 Luna pre column (2 × 4 mm) and C18 Luna column (2 × 150 mm, 100 Å, 3 μm) (Phenomenex, Torrance, USA). Mobile phases were 0.1% aqueous formic acid (pH 4.0; eluent A) and acetonitrile (eluent B). The following elution gradient was used: 10% B = 0.00–2.00 min; 75% B = 2.01–15.00 min; 90% B = 15.01–21.00 min; 10% B = 21.00–30.00 min. Sample injection volume was 10 μL at a flow rate of 0.2 mL min^−1^, with the temperature of the column maintained at 40 °C. Parameters for MS analysis were set using negative ionization mode with spectra acquired over a mass range from *m/z* 50–1200. The acquisition parameters were capillary voltage, 4.0 kV; nebulizing gas pressure, 2 bar; drying gas temperature, 180 °C; collision RF, 150 Vpp; transfer time 70 μs, and pre-pulse storage, 5 μs. Mass calibration was achieved using sodium formate (10 mmol L^−1^) over a mass range from *m/z* 50–1200. In addition, the automatic MS/MS experiments were carried out by adjusting the collision energy values as follows: *m/z* 100, 15 eV; *m/z* 500, 35 eV; *m/z* 1000, 50 eV, using N_2_ as collision gas.

Data from LC–MS/MS were processed using Data Analysis 4.0 software (Bruker Daltonics, Bremen, Germany). For the targeted evaluation, the identities of phenolic compounds 4-hydroxybenzoic acid, ferulic acid, and *p*-coumaric acid were confirmed with external standards (Sigma-Aldrich, São Paulo, Brazil) by comparing retention times (Rt), exact mass, and fragmentation and isotope profiles. Individual phenolic compounds were quantified by comparing their peak areas with those of calibration curves of each external standard. For the untargeted evaluation, peak detection was achieved with Profile Analysis 2.1 software (Bruker Daltonics, Bremen, Germany) using the Find Molecular Features (FMF) algorithm as follows: S/N threshold = 3; correlation coefficient threshold = 0.7; minimum compound length = 10 spectra; and smoothing width = 1. To generate a three-dimensional bucket table with *m/z*, Rt, and intensity of each feature detected, the *Advanced bucketing* was set with time and mass thresholds of 0.5 min and 5 ppm, respectively. The *Bucket filter* was set to generate a bucket table with 70% of the features of a group attribute within a bucket (susceptible and resistant cultivars).

### Research involving plants

The commercial rice cultivars (*Oryza sativa* L.) used in this study were provided by Embrapa Temperate Agriculture (Pelotas, RS, Brazil). This study complied with institutional and national guidelines for experimental research involving plants.

## Data analysis

Data from field screenings for antixenosis consisted of numbers of feeding scars and eggs (average from 5, 8, and 11 DAF) and data for antibiosis consisted of numbers and weight of larvae (average from 15, 25, and 35 DAF), TE50%, and weight adult offspring (average of males and females). These data were checked for normality by Shapiro–Wilk and homoscedasticity by Bartlett and analyzed by Analysis of Variance (ANOVA) with the Scott-Knott post hoc test (*P* ≤ 0.05) using the “ExpDes” and “easyanova” packages in the R software^72^. Using the hierarchical procedure based on the Euclidean distance matrix, a cluster analysis^73^ was done on the above-described antixenosis and antibiosis variables on tested cultivars. Dissimilarity in cultivar susceptibility to RWWs was determined based on the highest distance value obtained from the pairwise comparison between cultivars from the distance matrix.

Since this was a field study with uncontrolled natural infestation, in order to be able to compare biochemical responses of cultivars at the same level of larval infestation, root samples collected at 15, 25, and 35 DAF were classified in two distinct groups: I) samples that exhibited natural infestations of larvae equal or close to the corresponding average infestation for each cultivar (average of larvae at 15 DAF: BRS Pampa CL = 4.94; Dawn = 4.15; 25 DAF: BRS Pampa CL = 16.56, Dawn = 4.33; 35 DAF: BRS Pampa CL = 20.81, Dawn = 5.08); II) samples that exhibited natural infestations of larvae at the following levels: 1 = 0 larva (plants that did not show larval infestation were used as a control); 2 = 1–3 larvae; 3 = 5–7 larvae; 4 = 9 larvae. Thus, the biochemical analyses were conducted in the following way: (A) estimations of SOD, CAT, APX, POX, PPO, phenolic compounds, and LTGA on root samples from group I at each sampling time (15, 25, and 35 DAF) using a factorial scheme of 2 (cultivar) × 3 (sampling time); (B) estimations of SOD, CAT, APX, POX, PPO, phenolic compounds, and LTGA on root samples from group II at 25 DAF using a factorial scheme of 2 (cultivar) × 4 (infestation level); C) annotation of metabolites on root samples from group I by LC–MS/MS untargeted analysis, considering the differentially accumulated metabolites between resistant cultivar versus susceptible cultivar regardless of sampling times. For all cases, a completely randomized design was used with six replications consisting of one plant from each plot.

Quantitative differences in enzymatic activities, phenolic, and LTGA contents among cultivars of contrasting susceptibility for all sampling times or infestation levels were determined by ANOVA with the Scott-Knott post hoc test (*P* ≤ 0.05) using the “ExpDes” and “easyanova” packages in the R software^72^. The assumptions of normality and homogeneity of variance were tested for all variables by the Shapiro–Wilk and Bartlett tests, respectively. Log-transformation was applied to data that did not meet these premises.

Untargeted LC–MS/MS data analysis were performed using MetaboAnalyst 4.0 software^74^. Normalization by sum, Log-transformation, and Pareto scaling were initially applied to the whole LC–MS/MS-derived dataset in the data pre-treatment step. Orthogonal Projections to Latent Structures Discriminant Analysis (OPLS-DA) supervised method was used to determine the metabolite separations in each group (resistant cultivar versus susceptible cultivar). The statistical robustness of the OPLS-DA models was assessed by examining the goodness of fit (R^2^Y = close to 1) and the predictive ability of the models (Q^2^ = 0.50–1.00). For this, Permutation tests were performed with 1000-fold repetition (*P* ≤ 0.05). Significant differences in metabolites among cultivars of contrasting susceptibility were determined by Student’s t-test (*P* ≤ 0.05) and Fold Change (FC) of ≥ 2.0. The false discovery rate (FDR) was calculated to adjust *P*-values. Significant features from LC–MS/MS untargeted analyses were tentatively identified (annotated). The elemental composition of each annotated compound was selected according to accurate masses and isotope profiles using *Smart Formula* (DataAnalysis 4.2, Bruker Daltonics, Bremen, Germany), which provides a list of possible molecular formulas with their respective error (ppm) and mSigma (isotope profile similarity index between predicted and experimental values). Compound annotation was performed by matching the accurate *m/z*, isotopic profile, and MS^n^ fragmentation patterns with data from databases (FoodB, HMDB, MassBank, METLIN, and PubChem) and reference literature using 10 ppm of accurate mass precision.

## Supplementary Information

Supplementary Information.

## Data Availability

All data generated or analyzed during this study are included in this published article and its supplementary information file.
