# Supplementary material for: Morphoanatomical and biochemical factors associated with rice resistance to the South American rice water weevil, Oryzophagus oryzae (Coleoptera: Curculionidae)
Source: Sci Rep. 2022 Dec 28;12:22480. doi: 10.1038/s41598-022-27080-3 (PMC9797491; doi:10.1038/s41598-022-27080-3)
Supplement: Supplementary file 1 — Supplementary Information. [file 41598_2022_27080_MOESM1_ESM.pdf]

Morphoanatomical and biochemical factors associated with rice resistance to the South American rice water weevil, *Oryzophagus oryzae* (Coleoptera: Curculionidae)

Scientific Reports

Juliano de Bastos Pazini*, José Francisco da Silva Martins, Keilor da Rosa Dorneles, Rosane Lopes Crizel, Fernando Felisberto da Silva, Fábio Clasen Chaves, Juliana Aparecida Fernando, Leandro José Dallagnol, Enio Júnior Seidel, Michael Joseph Stout, Anderson Dionei Grützmacher

*Corresponding author: Federal University of Pelotas, Department of Plant Protection, Postal Code: 96160-000, Pelotas, RS, Brazil. Current address: University of São Paulo, Department of Entomology and Acarology, Postal Code: 13418-900, Piracicaba, SP, Brazil. E-mail: julianopazzini@hotmail.com. Phone: +55 55 99934-6181

Supporting information

Include:

Table S1: Rice cultivars assessed in field screenings for host plant resistance to the South American rice water weevil (RWW) *Oryzophagus oryzae* at the seasons 2016/17 and 2017/18.

Figure S1: Volcano plot with metabolites that were significantly accumulated between the resistant rice cultivar to the South American rice water weevil *Oryzophagus oryzae* compared to susceptible one.

Table S1. Rice cultivars assessed in field screenings for host plant resistance to the South American rice water weevil (RWW) *Oryzophagus oryzae* at the seasons 2016/17 and 2017/18.

Plant material	Cultivar type[1]	Plant type	Cycle[2]	Year Released[3]	Origin[4]
BRS Atalanta	CI	Philippine-modern	VS	2004	Brazil[#]
BRS Firmeza	CI	American-modern	S	1999	Brazil[#]
BRS Ligeirinho	CI	Philippine-modern	VS	1995	Brazil[#]
BRS Pampa CL	HTI	Philippine-modern	S	2016*	Brazil[#]
BRS Querência	CI	Philippine-modern	S	2005	Brazil[#]
Dawn	CI	Intermediate or American	M	1970	United States[$]

[1]CI= Conventional inbred; HTI= Herbicide tolerant inbred. [2]Rice cycle: VS: Very short= <100 days; S: Short= 110 - 120 days; M: Medium= 121 - 130 days. [3]*2016: year of release for scientific studies (2019: official year of release for trading). [4#]Brazilian Agricultural Research Corporation (EMBRAPA); [$]Beaumont Research and Extension Center.

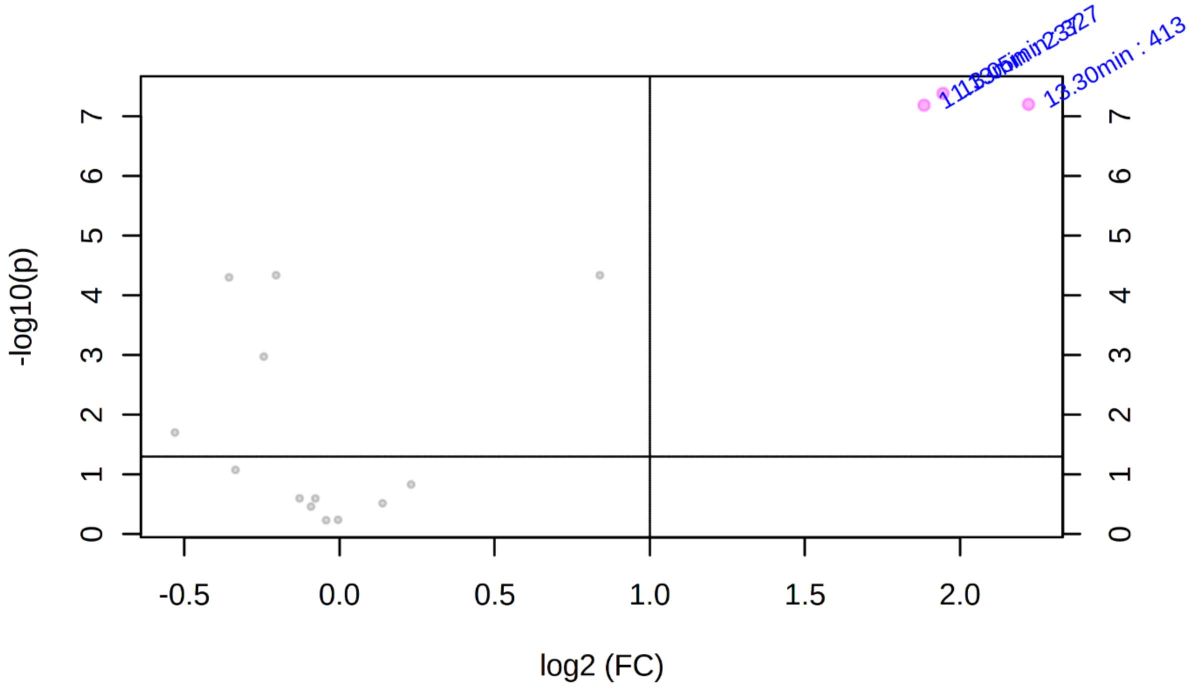

Figure S1. Volcano plot with metabolites that were significantly accumulated between the resistant rice cultivar (‘Dawn’) to the South American rice water weevil *Oryzophagus oryzae* compared to susceptible one (‘BRS Pampa CL’). Volcano plot with metabolites that were significantly accumulated between the resistant rice cultivar (‘Dawn’) to the South American rice water weevil *Oryzophagus oryzae* compared to susceptible one (‘BRS Pampa CL’).The red dots represent metabolites above the threshold. The further the metabolite’s position away from the zero (0, 0), the more significant the metabolite is. Volcano plot visualizes the -$\log_{10}$ adjusted *P*-values and $\log_2$ Fold-Changes (FC). Metabolites with adjusted *P* less than 0.05 and a FC greater than 2-fold are highlighted and labeled.
